# A qualitative study of the allergy testing experiences, views and preferences of adult patients

**DOI:** 10.1186/s13601-016-0125-8

**Published:** 2016-08-30

**Authors:** Clare E. Brown, Christina J. Jones, Laura Stuttaford, Annalee Robertson, Rabia S. Rashid, Helen E. Smith

**Affiliations:** 1 Division of Primary Care and Public Health Medicine, Brighton and Sussex Medical School, Room 319, Mayfield House, Village Way, Brighton, BN1 9PH UK; 2 Department of Respiratory Medicine, Royal Sussex County Hospital, Brighton, UK

**Keywords:** Allergy, Allergy testing, Patient preferences, Adult, Skin prick testing, Blood tests

## Abstract

**Background:**

Patients can be tested for IgE sensitivities with in vivo or in vitro testing, but patients’ experiences of different allergy test modalities have not been studied. The objective of this study was to investigate adult patients’ experiences, views and preferences for allergy testing, exploring skin prick testing and allergen-specific IgE testing.

**Methods:**

A qualitative study of adults attending out-patients for investigation of a suspected allergy. A purposive, convenience sample identified participants and semi-structured interviews were conducted, face to face or by telephone. Interviews were recorded and transcribed verbatim. Thematic content analysis was used to explore patients’ experiences.

**Results:**

23 patients were interviewed. The characteristics of skin prick tests particularly valued were the immediacy and visibility of results, which enabled testing and interpretation to be achieved within a single clinic appointment. In vitro testing offered patients simplicity and procedural speed, necessitating only a single puncture site, and was perceived to be a superior test as it was conducted in a laboratory.

**Conclusions:**

The patient preferred method of allergy testing was skin prick testing rather than in vitro allergen specific IgE testing. However, most patients were accepting of either testing modality because their desire to confirm or exclude an allergic trigger overrode any perceived disadvantages of the test method.

**Trial registration:**

Not applicable as study descriptive and qualitative.

## Background

Allergy diagnostic tests are used as adjuncts to the clinical history and physical examination in formulating the diagnosis for the patient. Two most commonly used methods of confirming IgE-mediated sensitisations are in vivo percutaneous skin tests (SPT) or in vitro specific IgE testing. SPT involves placing a drop of allergen reagent onto the skin of the patient and using a 1 mm lancet to puncture the epidermis. A positive response is indicated by the development of a wheal and flare response. The serum allergen specific IgE test involves the standard venepuncture procedure with serum IgE levels being analysed by immunofluorescence-based immunoassay (ImmunoCAP). The SPT is the gold standard, however the serum allergen specific IgE test is used when patients have severe dermatographism or are unable to stop taking antihistamines [1].

Whilst patient’s perceptions of pathways to and accessibility of allergy testing, have been investigated in one previous study [2], no study has addressed patients’ experiences and preferences of test modality, despite a growing need to consider this. Currently there is a move towards providing health services which are patient led. In the UK a recent NHS reform programme, ‘Creating a Patient Led NHS’, challenges us to move away from the traditional, rather paternalistic health service that ‘does things for its patients’ and move towards one that is patient led in its organisation and delivery [3]. Addressing patient’s experiences enables the development of patient-focused care which in turn improves satisfaction, health outcomes and the physician’s performance [4]. Using qualitative methods, we were able to gain insight into the experiences and views of adult patients undergoing allergy testing and the reasons for their expressed preference for in vivo skin prick testing or in vitro serum allergen specific IgE testing.

## Methods

Due to the study’s exploratory nature of a health topic that has received little previous investigation a qualitative approach was used with one-to-one interviews.

### Patient recruitment

Adult patients, 17 years old and above, were recruited from an Allergy outpatient clinic in a teaching hospital in the south east of England. Patients were eligible if they had undergone any allergy testing in this clinic in the preceding 8–10 weeks. Purposive sampling, rather than random sampling, was used to ensure we had informants from a wide age range. Interested patients were provided with verbal and written information about the study before written consent was obtained.

### Interviews

In-depth interviews were conducted, face to face or by telephone, by three researchers (LS, CB & AR) independent of allergy service provision and trained in qualitative methods. A topic guide developed, with input from clinical colleagues, to shape the conversation around the patient’s experiences of allergy testing, probing to understand the expressed positive and negative experiences. If interviewees had experience of both in vitro and in vivo allergy testing they were asked to compare and contrast their experiences. If a participant had only undergone one mode of testing they were given a standardised description of the other test procedure and asked to reflect on that. This enabled all participants to comment on their preference for mode of allergy testing. Interviews were recorded and transcribed verbatim.

### Data analysis

The transcripts were analysed using Burnard’s method, a very structured and rigorous approach for thematic content analysis [5]. To ensure reliability data were initially analysed by each researcher to independently develop a coding framework, these were then compared and discussed to develop an analytical framework with eight themes. Results are illustrated with selected quotes to illustrate the theme being discussed; the gender and age of the respondent are shown in parenthesis.

## Results

Twenty-three interviews were conducted. Respondents ranged in age from 17 to 77 years (mean 40, mode 31), 13 (56.5 %) were females. Fourteen respondents had recently experienced SPT, seven patients had received both a SPT and specific IgE blood test and two had undergone the blood test only. Eight patients had experience of allergy testing in the past and all patients had undergone venepuncture before (Table 1).

**Table 1:** Demographic and clinical characteristics of adult participants

ID	Age	Sex	Patient preference SPT versus blood test	Occupation	Presenting problems	Previous allergy testing? (details, if known)	Recent allergy testing
A1	17	M	SPT	Student	Rhino-conjunctivitis	Yes (SPT)	Blood test
A2	27	F	No preference	Credit control analyst	Suspected sea food allergy	Yes (SPT)	SPT
A3	31	M	SPT	Project manager	Seasonal rhinitis Food allergies (egg and nuts)	Yes (SPT & Blood test)	SPT & Blood test
A4	23	F	Blood test	Secretary	Venom anaphylaxis	No	Blood test
A5	31	M	Blood test	Financial advisor	Churg-Strauss syndrome –rhinitis, polyps	No	SPT
A6	57	M	SPT	Director of engineering company	Angioedema	No	SPT & Blood test
A7	31	M	SPT	Manual labourer	Perennial rhino-conjunctivitis	No	SPT
A8	31	M	SPT	Barber	Perennial and seasonal rhino-conjunctivitis	Yes (SPT)	SPT & Blood test
A9	77	F	SPT	Retired	Suspected latex allergy	No	SPT
A10	32	F	SPT	Accounts manager	Seasonal Rhinitis Oral allergy syndrome	No	SPT
A11	40	F	SPT	Freelance journalist	Seasonal and perennial rhinitis	No	SPT
A12	75	F	SPT	Retired	Suspected food allergy	Yes (Blood test)	SPT
A13	43	F	SPT	Graphic designer	Seasonal and perennial rhinitis	No	SPT
A14	48	F	SPT	Children’s social worker	Seasonal rhino-conjunctivitis	No	SPT
A15	44	F	Blood test	Nurse	Perennial rhinitis	No	SPT
A16	60	F	SPT	Nurse	Angioedema	Yes (SPT & Blood test)	SPT
A17	22	F	SPT	Student	Asthma triggered by cat exposure	No	SPT
A18	42	F	SPT	Media/PR agency (3)	Asthma and rhinitis Suspected sea food allergy	No	SPT & Blood test
A19	28	M	SPT	Chef	Seasonal rhinitis Oral allergy syndrome	Yes (SPT)	SPT
A20	31	M	SPT	Political campaign officer	Perennial rhino-conjunctivitis	No	SPT
A21	23	M	SPT	Retailer	Difficult to control asthma	No	SPT and Blood test
A22	41	M	Blood test	Painter and decorator	Asthma, suspected dog allergy	No	SPT & Blood test
A23	58	F	No preference	Voluntary financial work	Anaphylaxis, suspected nut allergy	Yes (SPT)	SPT & Blood test

### Understanding of skin prick testing procedure

Patients’ knowledge of skin prick testing prior to their outpatient consultation was variable, mainly uninformed, some admitted to having ‘never heard about it’, or if they had had one previously, not remembering the detail. Where they did recall recent information, relatives and friends were the main sources, and the information was often partial:

‘Well I’d only ever heard about the, the, when they put the thing on your forearm. I didn’t realise it was a pinprick, I though they just put a bit of solution, they put on there and done you that way.’ (Male, 41)

‘I knew that I was going to have little drops on my arms, but I didn’t know that they were going to scratch the surface ……’ (Female, 43)

Other patients were misinformed or had misconceptions:

‘I thought someone was going to scrape a bit of my skin off and take a bit of blood, that’s what I thought.’ (Male, 31)

### The SPT procedure

The SPT procedure was fluently recalled:

‘Marked my arm with that strip and then put the drops of each thing on and then used the needle to prick the skin and then sat me in the waiting room. Waited for a while and then called me into check whether I had reacted to anything, which I hadn’t….’ (Male, 31)

The SPT procedure was described largely as acceptable, the descriptors used ranged from ‘no problem’ or ‘not painful’ through ‘fine’ or ‘ok’ to ‘very good’ or ‘perfectly comfortable’. ‘It didn’t hurt’ was a very common descriptor, for example ‘you know I was a bit anxious about it, because I am not very good with pain generally, (laughs) but actually it was absolutely fine, it didn’t hurt it was fine.’ (Female, 43)

Two respondents mentioned the anxiety generated by learning their skin would be pierced, but the reality was not problematic, ‘…it just felt like getting a pine needle.’ (Female, 22)

However, there were some who described their SPT in more negative terms:

‘it was like I’d been bitten by a million ants or something, it was pretty intense, having 10 needles all at once, but it wasn’t unbearable, it was you know, I knew it was going to happen.’ (Female, 32)

‘well I think that was the most, the most discomfort I’ve had, being in the allergy clinic, that part, it, I thought I didn’t like it at all.’ (Male, 41)

One person described his concern that not all the pin pricks were equal:

‘I was a bit concerned sometimes when the skin was being pricked. I was a bit concerned as to whether some of those things that you use to prick were pricky enough. I felt some of them and I didn’t feel some of the other ones. So some were actually I could feel them pricking me where others I didn’t feel prick me and I almost thought that it wasn’t done hard enough, if that makes sense?

So my trust in the process was a little bit wary.’ (Male, 31)

He was therefore worried that there would be potentially false negative results where there was no sensation of the lancet piercing his skin. As it happened, some of those tests where he was not sure the lancet had pierced the skin enough did react, so he was reassured. However had his results been negative he may not have been convinced of the accuracy of the results.

### Longer term discomfort

In their accounts of their experiences of skin prick testing respondents distinguished between the sensations of skin pricking and the subsequent discomfort whilst waiting for the results to be read:

‘You know it’s not really nice feeling, its irritation obviously.’ (Male, 28)

‘It, it was fine. I mean it was really itchy afterwards, but the actual test was fine’ (Male, 23)

Only one person described prolonged discomfort, saying ‘that skin prick test one, it made my arm itch for days after’ (Male, 41)

### Interpretation of SPT

Several aspects about the experience of acquiring the SPT results were commented on, including speed, visibility and impact. The rapid availability of the SPT results was greatly valued, respondents described the results as being ‘instantaneous’, ‘in real time’ or ‘immediate’, ‘You know within 15 min you’ve got the results.’ (Male, 31)

Another positive aspect was the visibility of the emerging response and comparing the size of the different reactions, ‘We sat there all waiting to see if we reacted to it ….you can physically see as a patient.’ (Male, 31). This visibility was described with adjectives such as ‘interesting’ and ‘informative’, but also as ‘empowering’ and ‘engaging’.

‘I liked the fact that you could see what you reacted to so it was very obvious that you had a reaction to them rather than someone just telling you, you are allergic to something’. (Male, 31)

Not being entirely dependent on a third-party and being able to ‘confirm that for my own eyes’ was perceived as a powerful aide to patient understanding and as a prompt to action:

‘That was quite a good way of illustrating the allergy.’ (Male, 31)

‘I reacted quite badly to the dust….blatantly obvious how bad it was, and it prompted us to rip up our carpets for example, and get laminate flooring so it doesn’t hold the dust in the room, and that kind of thing.’ (Male, 23)

### Opportunity to discuss test results

Clinician interpretation of the results 15 min later generated a valued opportunity to discuss the implications of the findings within the same outpatient visit rather than having to wait to be informed by post or return for a follow up appointment. Despite these face-to-face discussions some misunderstandings were highlighted, for example one patient recalled the explanation of his Pollen Food Syndrome as contamination with pollen rather than cross- reaction with other food proteins:

‘….I’m increasingly reacting to fruits and hazelnuts, um skin tests didn’t detect that I was allergic to the, um because I’m allergic to pollen the suggestions was that the pollen gets into the fruit and the hazelnuts and that could cause me to react, so um that’s kind of useful information.’ (Male, 31)

The interpretation of a wheal as sensitisation not allergy was also confusing to some. One patient asked:

‘Does that mean I am a little bit allergic to salmon, to cod, to the, to the, to the, but I mean I eat that on a daily basis.’ (Female, 27)

### In vitro testing experiences

#### Blood taking process

When talking about their experiences of in vitro allergy testing respondents focussed on the venepuncture procedure and there was limited awareness of what the test was assessing or what happened to the sample in the laboratory. Patients referred to it generically as ‘a blood test’ and commented on comfort and speed, for example

‘It was fairly good, fairly fast and it didn’t hurt or anything.’ (Male, 17)

‘It didn’t take longer than 2 min.’ (Male, 31)

Some respondents described a degree of physical pain, ‘not too bad’, ‘just mild discomfort’. Discussing their venepuncture for in vitro allergy testing prompted memories of previous blood sampling, respondents often digressing to recall less than optimal experiences of locating a vein or the resultant bruising. Some respondents described their coping strategies for venepuncture ‘I look away and don’t watch the needle go in’ (Male, 31). Others alluded to emotional rather than physical discomfort, ‘No forewarning, I was wearing a top that I had to take off’ in an area she perceived as communal. (Female, 58)

#### Delay receiving blood test results

Whilst the speed of venepuncture was noted respondents also talked less favourably about the time to obtaining results

…it doesn’t take much time, but with the blood test it take a few weeks to get the results back.’ This respondent went on to comment on the impact of the results ‘….and you don’t see it for yourself so you’re obviously relying on someone else to you know, make the judgement about whether you reacted to it. …. there’s still that slight doubt in my mind that I may react to them…. Information can get lost in translation.’ (Male, 31)

However, this delay was not **always** perceived as negative, one lady described how it gave her time to acclimatise ‘…it was all quite new and frightening so I wasn’t really in a hurry [to know]….’ (Female, 23).

People had different experiences of learning of their IgE results, some in person, some written ‘letter in the post…with a paragraph about the blood test’ and some were still waiting.

### Perceived strengths of the different test modalities

Characteristics frequently listed in favour of SPT included minimal discomfort, immediacy of the results and the opportunity to see the results oneself (‘it really did flare up….quite substantially. Whereas ……. a blood test, you don’t see it’ (Male, 17)) and to discuss the results. The attributes of IgE in comparison to SPT were single puncture site (‘I think a blood test would be better. Because it’s just a prick.’ (Female, 44)) and minimal patient involvement.

Respondents also talked about their perceptions of blood testing as being more technical and having greater discerning powers:

‘I mean the prick test, as I said, it’s a very simple one, and the blood test is probably a more in depth test.’ (Female, 27)

‘I think that a blood test is more accurate because it’s kind of part of you, more than just a few dots on a skin prick test. I think it can find out really what is wrong with you.’ (Female, 23)

This perception of superiority of in vitro testing was sufficient for some interviewees to reflect on their willingness to tolerate pain or overcome needle phobia for a better test:

‘I mean I hate blood tests, but if having a blood tests means that I would know what definite, you know I am allergic to then I will do it.’ (Female, 27)

Three respondents (two nurses and one financial adviser) spontaneously brought up issues relating to the efficiency of the two testing modalities, making a comparison between ‘one injection’ and the very visible multi-stage process of SPT with lancet pricks, waiting, and clinical interpretation and feedback. They wondered whether venepuncture was ‘more efficient from the NHS’ point of view’. One respondent commented:

‘…. [in vitro testing] may be cost saving, to give a vial of blood that goes off, to haematology or whichever department will look at it, and they’ll go do their tests. And so you haven’t got loads of people running round measuring things, pricking arms, sending you back into the waiting room, sending you back in again.’ (Female, 60)

Qualitative research does not generally seek to enumerate, but at the close of the interview respondents were asked to comment on their preference for allergy test modality. Some patients were very decisive; others hesitated before coming to a conclusion, explaining that their desire to confirm or exclude an allergic trigger may override any perceived disadvantage of the allergy test.

‘So I wouldn’t mind either of them to be honest. As long as it does, you know it does tell me what is my problem.’ (Female, 27)

Seventeen of the 23 patients finally expressed a preference for SPT and four for in vitro testing. The remaining two had ‘no strong feelings either way’ and perceived the two testing modalities as equivalent. Of the ten participants who had experience of both in vivo and in vitro allergy testing, eight expressed a preference for skin prick testing, one for venepuncture and one expressed no preference. This distribution of expressed preferences was mirrored in those who had only theoretical knowledge of one or other of the allergy testing modalities.

## Discussion

Exploring patients’ experiences and preferences for allergy testing in open ended interviews demonstrated many positive views about SPT, its valued attributes including the immediacy of the results and the opportunity to see the results for one’s self. From the interviewees’ perspective in vitro testing offered speed and simplicity of patient involvement, single puncture site, and no residual side effects, such as itching. SPT was the preferred method of allergy testing for the majority of patients. Without any understanding of what happened to the blood sample once taken, in vitro testing was perceived as technically superior and it was this assumption that sometimes led to patients’ ambivalence of testing modality or an expressed preference for a blood test.

### Strengths and weaknesses

There are studies comparing the performance characteristics of in vitro and in vivo tests (sensitivity and specificity), but this is the first in-depth qualitative study investigating patients’ experiences, and views of allergy testing. The semi-structured interviews enabled participants to raise issues important to them and produced rich information covering many aspects of the allergy testing process in an outpatient setting. We took steps to increase the validity of our data, including the use of interviewers with no clinical expertise in allergy in order to reduce the possibility of contamination of lay beliefs with professional views and knowledge. A systematic and fully documented approach to data analysis was undertaken by three researchers independently to minimise researcher bias. Our purposive sampling achieved a sample with a range of ages, socioeconomic backgrounds and clinical conditions in which we could explore similarities and differences in views. With its narrow aim, high specificity (participants had all recently undergone allergy testing) and the focused dialogue this study would be categorised as a qualitative study with high information power [6]. Validity was further ensured by continuing sampling until data saturation was achieved, that is the point at which three consecutive interviews did not reveal any further themes. Recruiting patients from a single institution could be considered a weakness of the study, but interviewees drew on their experiences of allergy care from other health care settings, together with experience of venepuncture for other medical problems. As the data were collected in the context of the British National Health Service where care is free at the point of delivery, some caution may be required in generalising our observations on preference to systems where patients pay for their healthcare and where differential personal costs may influence choice. The data about the actual allergy testing experience is more generalizable, but inevitably there are context specific factors which influence responses, for example in our hospital it is the physician who performs and reads the SPT, but a phlebotomist takes the blood samples.

### Implications for clinical practice

Patients’ perspective reflects the anecdotal professional perspective ranking percutaneous testing first, but their reasons are different. Clinicians see the advantages of SPT as being minimally invasive, having good reproducibility, being easily quantified, and allowing the evaluation of multiple allergens at one session. From a clinical perspective in vitro tests are used when in vivo testing is contraindicated, as in patients with extensive dermatoses (e.g. atopic dermatitis or dermatographism) or patients unable to discontinue their antihistamines. We asked respondents to express their preferences but we recognise that there may not always be a patient choice (for example, a patient needing testing for venom allergy or component based diagnosis in the assessment of cross-reactivity or the prediction of severe, persistent symptoms), or when one test method is inappropriate or contraindicated. In some situations choice may be limited by availability or resource, for example where allergy testing is used so infrequently that having a range of allergens with l**i**mited shelf lives is not justifiable. Interestingly patients were sometimes bemused with the concept of choice in health care and verbalising their preferences, ‘It’s one of those things, you got to have it done, and you know you just accept that that’s the method to find out what you are allergic.’

An unintended benefit of this study was thorough feedback on our allergy service; patients commented that they did not always get a copy of their SPT results; the disruption of moving between different clinical areas for consultation, SPT and waiting; interruptions of their clinician by other staff. Our study also highlighted some weaknesses of patient–practice communication, for example when the allergen raised a wheal, but not one of sufficient size to be interpreted as a positive test result. Such patient misunderstanding of test positivity could have significant long term implications for their diet, activities and quality of life.

### Comparison with other studies

There has been much research into patient perceptions of the generic process of venepuncture, but no study has focussed on serum allergen specific IgE testing. Compared to SPT our participants had relatively little to say about in vitro testing. Interestingly this reticence to talk about venepuncture has been observed in another study exploring what people know and think about venepuncture and blood. Pfeffer et al. [7] found that their participants had very little to say about blood tests because venepuncture is widely understood as something ‘they just do in hospitals’. It is accepted without questioning, and is not perceived as worthy of discussion as it is everyday practice, and one which patients submit to by common consent, as de Certau describes, it is the ‘law of the place’ [8].

New skin prick testing devices are being developed with recent growth in multi-headed devices which allow for the application of several antigens simultaneously. New devices have been subject to head-to-head comparisons with existing devices for intra-device variability (wheal and flare sizes, sensitivity and specificity). Advertising of diagnostic devices focus on physician preferences and the patient’s perspective is rarely addressed, but one study comparing eight commonly used skin test devices (four multi-headed and four single devices) measured patient discomfort using the Wong-Baker FACES pain rating scale [9]. Pain was assessed only within seconds of the application of the skin test to minimise the influence of histamine on pain perception. The mean pain score for all devices tested ranged from 0.88 to 2.04 out of 10. These scores are considered to represent mild pain and our qualitative data are consistent with these quantitative observations.

## Conclusions

Diagnostic testing and allergen recognition is an essential part of the evaluation of a patient with suspected allergy. This naturalistic study provides insight into patients’ experiences and views of allergy testing. Many patients expressed a preference for skin prick testing, but this preference was not universal.

## Abbreviations

SPT: skin prick test
NRES: National Research Ethics Service
ELISA: enzyme-linked immunosorbent linked assay
IgE: immunoglobulin E
